# Voluntary medical male circumcision and educational gradient in relation to HIV infection among sexually active adult men in Eswatini: evidence from the national surveys in 2006–2007 and 2016

**DOI:** 10.1093/inthealth/ihad070

**Published:** 2023-09-13

**Authors:** Bongani Zakhele Masango, David Ferrandiz-Mont, Chi Chiao

**Affiliations:** HIV Program (HIV Prevention), Eswatini Ministry of Health, Mbabane H100, Eswatini; Public Health Surveillance and Emergency Response Department of Vallès Occidental and Vallès Oriental, Public Health Agency of Catalonia, Sant Cugat del Vallès, Barcelona 08173, Spain; Institute of Health and Welfare Policy, College of Medicine, National Yang Ming Chiao Tung University, Taipei 112, Taiwan, ROC; Institute of Public Health, College of Medicine, National Yang Ming Chiao Tung University, Taipei 112, Taiwan, ROC

**Keywords:** voluntary medical male circumcision (VMMC), educational gradient, Eswatini, HIV, sexual behavior

## Abstract

**Background:**

To address knowledge gaps, this study examined social determinants, such as education attainment and HIV prevention, among sexually active men (SAM), with a focus on voluntary medical male circumcision (VMMC).

**Methods:**

Two nationally representative surveys, the Eswatini Demographic and Health Survey 2006 and the Eswatini HIV Incidence Measurement Survey 2016, were used to estimate whether or not VMMC at the individual and community levels contributes to HIV disparities to any meaningful extent. Multilevel logistic regression models further explored the educational gradient in HIV infection for 2006–2007 and 2016 with regard to VMMC among SAM, while adjusting for household poverty, sexual practices and individual characteristics.

**Results:**

Among SAM with tertiary education, HIV prevalence declined from 25.0% in 2006–2007 to 10.5% in 2016. A 51% decrease in HIV prevalence was found to be associated with an increase in VMMC (adjusted odds ratio 0.49; 95% CI 0.40 to 0.60). Compared with SAM with tertiary education, those who had a lower level of education were more likely to have HIV infection and this education gradient effect had become particularly profound in 2016.

**Conclusions:**

VMMC began to be promoted in 2008 in Eswatini and results suggest its effect, along with the education attainment effect, significantly resulted in a meaningful reduction in HIV prevalence among SAM by 2016.

## Introduction

Despite the considerable progress made in combating the HIV pandemic, numbers of new HIV infections remain well above national and international targets^1–3^ thus making HIV prevention a key public health priority, particularly in southern and eastern Africa, where 20.7 million people were living with HIV in 2019 with 730 000 new infections in the same year.^4^ Again there has been immense pressure on low-income and middle-income countries (LMIC) caused by the decline in the financing of HIV-related services, with donors’ direction now focusing on the ever-rising non-communicable diseases such as cancers, diabetes and hypertension.^5,6^ According to a report by Joint United Nations Programme on HIV/AIDS (UNAIDS), resources for HIV responses in LMIC have been decreasing since 2018. As a result, none of the global targets set by UNAIDS for 2020 was met. Additionally, it was estimated that 3.5 million people contracted HIV and an additional 820 000 people died of AIDS-related illnesses between 2015 and 2020.^7^ LMIC have therefore been urged to step up and fund their own responses, thus slowly filling some of these funding gaps and working towards a more sustainable response to HIV that maximises effectiveness in order to achieve the global goal of epidemic control by 2030.^8,9^

Voluntary medical male circumcision (VMMC) has emerged as an effective intervention that reduces the risk of HIV acquisition among men. It is estimated to reduce the risk of female-to-male transition of HIV by 60%.^2,10–14^ There is some evidence that VMMC may also reduce the risk of other sexually transmitted infections (STIs), such as syphilis, herpes simplex virus type 2 and human papillomavirus (HPV).^15^ With VMMC being a one-time, efficient, safe and cost-effective HIV prevention method, both WHO and UNAIDS identified 15 African countries with high HIV prevalence and low male circumcision (MC) prevalence as priority countries to scale up VMMC.^6,16,17^ Eswatini has one of the lowest (8.2%) levels of MC among the 15 VMMC priority countries.^18^ In 2008, Eswatini developed the Policy on Safe Male Circumcision for HIV Prevention and the Strategy and Implementation Plan for Scaling Up Safe Male Circumcision for HIV Prevention 2009–2013.^19,20^ The main objective was to provide VMMC services to 144 688 HIV-negative males during the period 2009–2013. Males aged 15–24 y were selected as the primary population, being at greatest risk of HIV infection.^21^ In 2011, the country further launched the Accelerated Saturation Initiative, with the goal of circumcising 80% of males aged 15–49 y within 1 y. Circumcision rates rose from 8.2% in 2007 to 35% in 2018. Around 29% of young men (aged 15–24 y) and 17% of men aged over 25 y have been circumcised, with 95% of the circumcisions solely for HIV prevention.^16^ Growing evidence has shown the use of antiretroviral therapy (ART) for the treatment of HIV-1 to be one of the most popular ideas to reduce the transmission of HIV.^22,23^ This strategy has generated a plethora of mathematical models that, for the most part, predict success.^24,25^ Several ecological studies argued that the prevention benefit of ART can already be seen in a few communities where ART usage was prevalent.^26,27^ Eswatini has one of the highest rates of antiretroviral therapy coverage of 79% in sub-Saharan Africa and 68% of those treated have a suppressed viral load, which indicates a successful treatment.^28^

For an intervention to be effective, social determinants, such as education, play a major role.^29,30^ Education has a critical role to play in mitigating the effects of HIV/AIDS, as it provides knowledge that inform self-protection; fostering the development of a personally held, constructive value system; inculcating skills that will facilitate self-protection; promoting behavior that will lower infection risks; and enhancing capacity to help others to protect themselves.^31^ With nearly two-thirds of Eswatini living in poverty, there are major inequalities in a number of social factors; for example, education costs are far higher than families can easily afford. Finally, a high rate of unemployment leads to an underestimation of the value of education.^32^

Literature has focused on the individual-level factors associated with HIV intervention, including access to testing and loss to follow-up during adherence to pre-antiretroviral therapy (pre-ART), ART initiation and ART treatment.^33–35^ Nevertheless, the arena in which social relations are constructed and reconstructed and where social change occurs is the community and this remains relatively unexplored,^36^ particularly when exploring the proportion of VMMCs within a community. Recently UNAIDS^6^ has highlighted the importance of community across the treatment cascade and has underscored how essential community-wide systems are for motivating the use of HIV diagnosis services and treatment services. They have argued for an expansion of research in this direction.^37^ Eswatini, a country with the highest HIV prevalence in the world at 27% and an HIV incidence of 1.13% in 2017 among adults aged 15 y and above,^38^ has continued to witness new HIV infections. There were 7785 new HIV infections in 2018 and 7367 new infections in 2019.^39^ The country was therefore selected as 1 of the 15 VMMC priority countries. There are therefore still knowledge gaps that need to be filled notwithstanding prior HIV research. Specifically, these include the relationship between community HIV prevention interventions, such as VMMC and HIV status; these have clearly been explored relatively less often. Surprisingly, little research has investigated the relationship between HIV prevalence and circumcision taking into consideration various social determinants such as education and, even less often, simultaneously included community-level variables. Thus, the present research first assesses whether there was a decline in HIV prevalence from 2006 to 2016 in Eswatini among sexually active men (SAM) during the period of the implementation of the Plan for Scaling Up Safe Male Circumcision for HIV Prevention 2009–2013. Next, we sought to understand to what extent VMMC at an individual level and at a community level is associated with the likelihood of becoming infected with HIV. By such investigations, we also seek to bring particular attention to education attainment and hypothesise that a decline in HIV prevalence depends on personal education attainment, when sexual and protective practices are taken into consideration.

## Methods

### Study design and population

We used data from two nationally representative household surveys done before and after the adoption of VMMC as an HIV prevention intervention; these were the Eswatini Demographic and Health Survey (EDHS) 2006 and the Eswatini HIV Incidence Measurement Survey (SHIMS) 2016. These surveys include information such as factors related to HIV prevalence and incidence, MC status and various socioeconomic characteristics, including household wealth and education, sexual health-related outcomes including condom use, number of sexual partners and sexual debut. Both SHIMS and EDHS data were collected using a two-stage sampling strategy. In both data sets the sampling frame consisted of all households in the country based on the Swaziland Population and Housing Census at survey year. The first stage selected enumeration areas (EAs) (clusters) using a probability proportional to size method. The EAs were stratified using four geographical regions (Hhohho, Manzini, Shiselweni and Lubombo), with each EA defined by its rural/urban status. During the second stage, a sample of households was randomly selected within each EA, or cluster, by an equal probability method. EDHS was obtained from the publicly available MEASURE demographic and health survey (DHS) website (https://dhsprogram.com and SHIMS https://phia-data.icap.columbia.edu/datasets?country_id=2&category_id=9&year_id=2016) and SHIMS from the Eswatini statistics office. Further details about the data collection and sampling design are described in the survey reports.^18,38^ Data collection and analysis were performed under strict ethical standards. The study protocol for this study was reviewed and approved by the Eswatini Health and Human Research Review Board and Institutional Review Board (IRB) from Colombia University for the SHIMS survey as well as National Yang Ming Chiao Tung University in Taiwan (IRB number YM111003E). The present investigation focused on Eswatini sexually active males (15–49 y old). Our analytical sample was 5088 men, which consisted of 2618 men from 275 communities in the 2006 EDHS data set and 2470 men from 286 communities in the 2016 SHIMS data set.

### Procedures and measures

Our outcome variable of interest is lifetime history of HIV infection. After the interview, a blood specimen from each interviewee was collected for HIV testing and the biomarker results of this test were used in the present study. HIV status was categorised as either HIV-positive (code 1) or HIV-negative (code 0).

VMMC was our main exposure of interest. Respondents were asked the question: ‘Some men are medically circumcised and others are not. Are you voluntarily medically circumcised?’ coded as ‘1’ if the answer was yes, otherwise coded as ‘0’. Respondents were asked a follow-up question: ‘How old were you when you were circumcised?’ In addition to individuals as the unit of analysis, the community was also another unit of analysis. We created a community VMMC norm variable by averaging all surveyed men aged 15–49 y and measuring the proportion of voluntary early male medical circumcisions that had occurred at ages 13 y or younger among these men within the communities. Communities with 0% of men having VMMC were categorised as communities with a ‘low’ VMMC norm, while more than 10% were regarded as communities with a ‘high’ VMMC norm, and the reference group was the communities with a VMMC norm of more than 0% but less than 10% of men having MC. Another main focus variable was education attainment, which measured the highest level of education of an individual and was categorised into five groups (none, primary, secondary, higher and tertiary). Time was an important indicator in this study; a categorical variable was created to distinguish the data collected at two time points: 2016 coded 1 and 2006 coded 0. As suggested by prior research,^40^ we also considered three sexual/protective behaviors, namely lifetime number of sexual partners, early onset of sexual activity and condom use. We also included individual characteristics such as household poverty, which was derived from the household wealth index, which consists of five quintiles of asset-based measurements. Each household asset for which information was collected was assigned a weight or factor score generated through principal components analysis. The resulting asset scores were standardised with a mean of 0 and a standard deviation of 1. These standardised scores were then used to create the break points that defined wealth quintiles as: lowest, second, middle, fourth and highest. A household was considered poor if the wealth index fell into the lowest two quintiles. Other individual variables included the individual's place of residence, marital status, age and region.

### Statistical analysis

Data analysis was performed using Stata 16 and all analyses were weighted to adjust for the sample design. Bivariate analysis to determine whether there had been a decline in HIV prevalence from 2006 to 2016 among SAM during the period of the implementation of the Plan for Scaling Up Safe Male Circumcision for HIV Prevention 2009–2013 in Eswatini was conducted. To understand the extent to which VMMC at an individual level and at a community level is associated with the likelihood of having HIV infection, we next calculated the intraclass correlation coefficient (ICC) to verify whether an analytical strategy involving multilevel logistic models is appropriate. The ICC for being HIV-positive was 0.045 in 2007 and 0.046 in 2016, which suggests that around 5% of the total variation in HIV status can be explained by the communities in 2007 and this was about the same in 2016 (p<0.01). We then performed multilevel logistic regressions separately for each survey year, as shown in Appendix 1. Lastly, multilevel logistic regression modeling was employed with progressive adjustments of three models after combining the two cross-sectional surveys. The crude model attempts to determine independently the factors associated with HIV status in terms of time interval, circumcision status, the household wealth, education attainment, sexual practices and individual background characteristics of the surveyed sexually active males in Eswatini. Model 1 explored whether or not VMMC at an individual level and a community level contributes to disparities in HIV prevalence to any meaningful extent by adding an interaction term between education and the survey year. In addition to exploring the education gradient, Model 1 was further extended to form Model 2, which included the sexual and protective practices; this allowed us to explore how much such behaviors contributed to the differences in HIV-positive prevalence from 2007 to 2016.

## Results

Table 1 shows the distribution of HIV prevalence by sample characteristics among the sexually active adult males in Eswatini by survey year. HIV prevalence showed a decrease from 2007 to 2016 across the whole sample (from 27.80% to 24.65%), among circumcised (from 25.8% to 14.51%) and among those who practiced early circumcision (from 20.95% to 16.39%). The proportion of sexually active males who were circumcised increased over this period (from 9.60% to 27.8%), while the proportion of those who practiced early circumcision decreased. Turning to sociodemographic characteristics, education attainment increased during this period and showed a remarkable decrease in non-educated sexually active males (from 9.74% to 3.11%), as well as an increase in those with tertiary education (from 9.86% to 12.21%). In the latter group, a reduction in the HIV prevalence was found (from 25.25% to 10.50%). HIV prevalence also decreased among those who had urban residence and those who were single, divorced, separated or widowed, while it increased among married or cohabiting males. The p-values are provided in Appendix 2.

**Table 1.** Participant characteristics [percentage or mean (SD)] and HIV prevalence by sample characteristics among the sexually active adult males who took part in Swaziland (Eswatini) Demographic and Health Survey (EDHS, 2006–2007, N=2 470) and Eswatini HIV Incidence Measurement Survey (SHIMS 2, 2016, N=2 618)

	EDHS, 2006–2007		SHIMS 2, 2016	
	Total sample	Percentage HIV-positive	Total sample	Percentage HIV-positive
Male circumcision (%)	9.60	25.82	27.82	14.51
Early circumcision at aged 13 y or younger (%)	5.92	20.95	4.88	16.39
**Sexual behavior characteristics**				
Lifetime number of sexual partners				
1–2	25.27	10.42	55.28	23.11
3–4	24.62	27.95	14.35	18.55
5–9	25.46	30.85	12.11	28.66
10 and above	18.49	46.09	9.96	36.79
Unknown status	6.16	30.97	8.29	25.05
Age at first sex 15 y or younger (%)	15.01	18.85	11.85	18.89
Used a condom at last sex (%)	48.20	28.35	55.51	28.02
**Sociodemographic characteristics**				
Age [mean (SD), range: 15–49]	30 (8.94)		31.1 (8.68)	
Education attainment (%)				
No education	9.74	34.31	3.11	35.28
Primary	30.67	29.16	26.06	35.12
Secondary	26.07	27.06	24.60	25.97
High school	25.23	23.66	34.02	19.78
Tertiary	9.86	25.25	12.21	10.50
Household poverty (%)	29.23	30.51	37.98	28.37
Marital status (%)				
Single	52.26	16.73	56.95	13.79
Married/cohabiting	40.54	36.77	37.76	38.03
Divorced/separated/widowed	7.20	57.62	5.29	46.04
Urban residence (%)	31.29	30.98	31.53	24.93
Regions (%)				
Hhohho	27.58	31.47	31.13	23.81
Lubombo	20.97	29.26	19.61	29.83
Manzini	32.93	25.40	35.51	23.65
Shiselweni	18.52	24.94	13.76	21.75
HIV-positive prevalence (%)		27.80		24.65

Table 2 presents the multilevel logistic regression models that estimate the likelihood of being HIV-positive among SAM aged 15–49 y. The unadjusted model shows the bivariate associations of VMMC at the individual and community levels, year, education attainment, sexual practices and individual background characteristics with the risks of HIV infection. VMMC at both individual (OR=0.50; p<0.01) and community levels are significantly associated with lower odds of being HIV-positive. Subjects living in communities with a low VMMC norm had higher odds (OR=1.22; p<0.05) of contracting HIV compared with their counterparts living in communities with median early circumcision prevalence. Turning to the sexual practices, all of them were significantly associated with HIV status. Males who had sexual intercourse at an early age (15 y old or earlier) were less prone to being HIV-positive (OR=0.63; p<0.001), while those who used a condom at last sex (OR=1.34; p<0.001) and those having more than two partners during their lifetime were more likely to contract HIV. Finally, those with a lower education level than tertiary (no education, primary, secondary education and high school) had higher odds of being HIV-positive (OR=2.52; p<0.001; OR=2.42; p<0.001; OR=1.82; p<0.001; and OR=1.34; p<0.001, respectively).

**Table 2.** Multilevel logistic regression models of predicting positive for HIV of the sexually active men aged 15–49 y for Eswatini, 2006–2016

	HIV-positive					
	Crude model		Model 1		Model 2	
	cOR	95% CI	aOR	95% CI	aOR	95% CI
HIV trend (2006–2007 DHS omitted)						
2016 SHIMS 2	0.93	(0.81–1.07)	0.51*	(0.30–0.85)	0.61^§^	(0.36–1.04)
**VMMC**						
Individual VMMC	0.50**	(0.42–0.61)	0.52**	(0.42–0.64)	0.49**	(0.40–0.60)
VMMC norm^†^ (reference = median)						
Low prevalence: 0%	1.22*	(1.04–1.42)	1.09	(0.92–1.29)	1.13	(0.95–1.35)
High prevalence: >10%	0.96	(0.76–1.21)	0.89	(0.68–1.17)	0.94	(0.71–1.34)
**Individual/household SES**						
Education attainment (reference = tertiary)						
No education	2.52**	(1.82–3.49)	1.35	(0.87–2.10)	1.49^§^	(0.95–2.33)
Primary education	2.42**	(1.87–3.13)	1.84**	(1.27–2.67)	2.10**	(1.44–3.06)
Secondary education	1.82**	(1.40–2.37)	1.98**	(1.36–2.88)	2.07**	(1.41–3.03)
High school	1.34*	(1.04–1.75)	1.71**	(1.17–2.50)	1.79**	(1.22–2.63)
Year × Education attainment						
Year × No education			2.30*	(1.08–4.87)	2.12^§^	(0.98–4.55)
Year × Primary education			2.38**	(1.36–4.18)	2.28**	(1.28–4.04)
Year × Secondary education			1.78*	(1.00–3.16)	1.74^§^	(0.97–3.13)
Year × High school			1.56	(0.88–2.77)	1.55	(0.87–2.78)
Household poverty	1.30**	(1.13–1.50)	1.16^§^	(0.99–1.38)	1.16^§^	(0.98–1.38)
**Sexual practices**						
Lifetime number of sexual partners (reference = 1–2)						
3–4	1.33**	(1.10–1.61)			1.49**	(1.20–1.85)
5–9	1.69**	(1.41–2.04)			1.61**	(1.30–1.20)
10 and above	2.93**	(2.41–3.56)			2.69**	(2.14–3.37)
Unknown status	1.62**	(1.26–2.08)			1.58**	(1.19–2.11)
Used a condom at last sex	1.34**	(1.18–1.53)			2.02**	(1.74–2.34)
Age at first sex 15 y or younger	0.63**	(0.52–0.78)			0.82^§^	(0.65–1.03)
*Background controls*						
Age (y)	1.09**	(1.08–1.10)	1.08**	(1.07–1.09)	1.07**	(1.06–1.08)
Marital status (reference = single)						
Married/cohabiting	3.37**	(2.92–3.88)	1.57**	(1.31–1.87)	1.76**	(1.46–2.11)
Divorced/separated/widowed	5.92**	(4.61–7.62)	2.71**	(2.05–3.59)	2.72**	(2.04–3.62)
Urban residence (reference = rural residence)	1.14^§^	(0.98–1.33)	1.24*	(1.04–1.48)	1.20	(0.99–1.44)
Regions (reference = Hhohho)						
Shiselweni	0.98	(0.80–1.20)	0.88	(0.70–1.20)	0.85	(0.68–1.07)
Lubombo	1.01	(0.83–1.24)	0.99	(0.81–1.22)	0.95	(0.76–1.17)
Manzini	0.90	(0.74–1.10)	0.91	(0.74–1.13)	0.82	(0.66–1.02)
*Compared with previous models*						
Wald χ^2^						79.47**
Degrees of freedom						6

^§^ *p*<0.10; *p<0.05; **p<0.01. cOR, crude odds ratio; aOR, adjusted odds ratio; VMMC, voluntary medical male circumcision. ^†^The VMMC norm was computed by averaging all surveyed men aged 15–49 y and measuring the proportion of early male circumcision that had occurred at ages 13 y or younger among these men within the communities.

**Figure 1.**
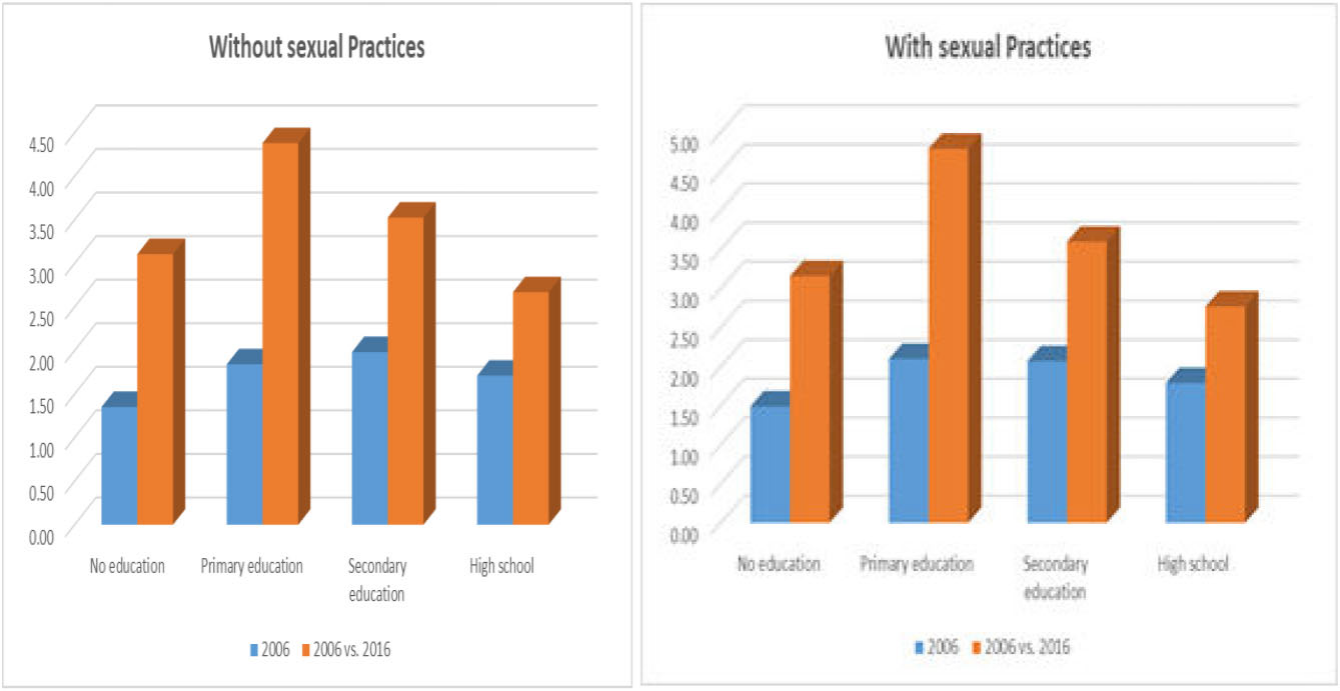
Adjusted odds ratios of education attainment on infecting HIV. Adjustments for VMMC norms, individual/household SES and background controls by year and separately for Model 1 (without sexual practices) and Model 2 (with sexual practices), and tertiary-level education as the reference group.

Model 1 attempts to disentangle the effect of circumcision on HIV status at an individual level and at a community level, as well as by the year of the survey by taking into account potential confounders and adding an interaction term into the model. After adjusting for background variables, MC remained consistently associated with HIV status whereas a low VMMC norm was no longer associated. Interestingly, the year of the survey now became significantly associated with HIV status, indicating a reduction in HIV prevalence over the studied years among tertiary educated males. Furthermore, the interaction term in the model was significant in that it revealed that the odds of being HIV-positive between 2006 and 2016 was contingent on the education level of the studied males. For example, SAM with no education were found to have HIV-positive odds that were 1.35 times higher than SAM with tertiary education; this effect appeared to be particularly significant in 2016 (when the effect was 1.35 × 2.30, that is 3.1); this is shown in Figure 1.

Model 2 added sexual practices into the model. While MC remains significantly associated with HIV status (OR=0.49; p<0.001), the year of the survey is now no longer significant. The interactive effect only remained significant among those males with primary education, thus indicating an increased risk of HIV infection among this group over the studied period compared with those with tertiary education. All sexual practices, with the exception of age at first sex being 15 y or younger, remain significantly associated with the studied outcome.

## Discussion

We wanted to examine the differences in HIV prevalence in 2006 and 2016 among sexually active males in Eswatini. There was a significant decline in HIV prevalence among sexually active males from 27.80% in 2007 to 24.65% in 2016. We further observed a significant decline in HIV prevalence among sexually active Eswatini males who were circumcised, from 25.82% in 2007 to 14.51% in 2016. One of the factors that could have contributed in the decline is the increase in circumcised males since the introduction of the local VMMC program. Another study in South Africa by Vandormael et al.^41^ found that, among circumcised men, the HIV incidence rate declined by 59%, from 1.24 (0.57 to 2.69) to 0.5 (0.16 to 1.57) events per 100 person-years between 2012 and 2016. These findings are biologically plausible: we would expect to observe earlier and larger population-level reductions in the male HIV prevalence following earlier VMMC scale-up. VMMC is beneficial for men and has been previously associated with a reduced risk of male HIV acquisition in sub-Saharan African settings.^10,21,42^ In 2009 a local VMMC program (soka uncobe) was introduced in Eswatini and, by 2018, self-reported circumcision coverage had reached 35%. Our results confirm the preventive benefits of circumcision for men, who have a lower incidence of infection when compared with uncircumcised men. The increase in circumcised males is likely to have contributed to the overall decline in HIV prevalence among sexually active male participants. We further explored whether or not MC at the community level contributes to disparities in HIV prevalence to any meaningful extent. Our findings provide evidence for a community association between early VMMC prevalence and HIV infection where respondents from communities with a low prevalence of early VMMC had higher odds (OR=1.22; p<0.05) of contracting HIV compared with their counterparts living in communities with higher early circumcision prevalence. Our findings are in line with those found by Cuadros et al.,^43^ where communities with low VMMC turned out to have high HIV prevalence, and these are the communities where a large proportion of the HIV epidemic is concentrated although the significance faded after controlling. It is therefore worth noting that MC could be influencing the community distribution of the HIV epidemic in Eswatini, as well as any local dynamics that are affecting the infection. Current evidence is suggesting that there are substantial declines in HIV prevalence in various parts of West, Southern and East Africa, which may support this hypothesis by the studies of Menon et al.^44^ and Sing et al.^45^ Furthermore, an educational gradient was further witnessed whereby those respondents with a lower education level than tertiary (no education, primary, secondary education and high school) had higher odds of being HIV-positive. Our study was in total agreement with studies^46,47^ which stated that more educated individuals have reacted more to information about HIV prevention and transmission and thus turn out to be less likely to be infected by the HIV virus.

Furthermore, HIV-positive status was associated with an increased number of lifetime sexual partners and use of a condom during their last sexual intercourse. This is in agreement with other studies done in the African context which state that the number of sexual partners increases one's risk of HIV exposure.^40,48,49^ The association between condom use and HIV positivity might be due to the fact that most of those reporting to have used a condom may have already been HIV-positive as studies in Kenya showed that people living with HIV (PLHIVs) were more likely to use condoms with primary sexual partners at last sex compared with secondary partners.^50^ Early sexual debut was also a protective factor for HIV status, which is in contrast to prior research in most sub-Saharan countries and South Africa.^51,52^ One of the possible reasons for this association may be the fact that these were men who, even if they began engaging in sexual intercourse younger, started with women of their own age who had lower risks of exposure to HIV.

Lastly, our study has in particular brought attention to the differences in 2007 and 2016 regarding HIV-positive status while taking into consideration education attainment. Our study pinpointed that there was a significant interactive effect between education level and year of survey. SAM with no education were found to have HIV-positive odds that were 1.35 times higher than SAM with tertiary education; this effect related to having no education appeared to be particularly significant in 2016 (when the effect was 1.35 × 2.30, that is 3.1). Studies over the years have demonstrated that education has a critical role to play in mitigating the effects of HIV/AIDS; it does so by providing knowledge that will inform self-protection. This happens via the fostering of the development of a personally held constructive value system, by inculcating skills that will facilitate self-protection, by promoting behavior that will lower infection risks and by enhancing capacity to help others to protect themselves.^31^ The argument is that education promotes both logical and significantly different ways of thinking, which allow better-educated individuals to take action to protect their health; as a result of their investment in their future, better-educated individuals have stronger incentives to protect their health.^53^

After adding sexual practices, the lifetime number of sexual partners that significantly impacts the HIV difference over the years is seen to be reduced and to become non-significant. Our results agree with other studies demonstrating that risky behavior is the most important factor associated with the prevalence of HIV. Our results are similar to findings from other analyses of DHS data and various cohort studies. Further analysis of nationally representative surveys from the Ethiopia DHS surveys in 2005 and 2011, the Malawi 2010 DHS and the Ghana 2011 DHS show the lifetime number of sexual partners to be one of the main predictors of HIV seropositivity.^54,55^

Our study had some strengths and limitations. First, our study uses self-reported measures of sexual behaviors and these may suffer from recall bias and social desirability bias. Second, the unavailability of some important variables of interest, such as the lifetime use of condoms and the consistent use of condoms, that were not included in this analysis made it difficult to ascertain condom use, as the prevention by using condoms is dependent on correct and consistent use. Third, while the clinical test for the presence of HIV is a valid outcome, nevertheless the fact remains that it is difficult to ascertain whether participants have become infected with HIV before circumcision or after circumcision among the older study subjects. Furthermore, this study did not adjust for HIV treatment as variables related to antiretroviral therapy and viral load were not available in the 2006-2007 EDHS dataset, we cannot control for treatment as prevention, as evidence has emerged supporting the fact that it is one of the growing prevention methods to reduce HIV incidence and Eswatini is achieving well in viral load suppression to reach the 90–90–90 UNAIDS targets. In spite of the above limitations, our study has important strengths. It uses biomarkers for the HIV status rather than self-reported status and thus it avoids information bias. Furthermore, the study used a relatively large recent nationally representative sample in two different points of time, and further adjusted all of the various analyses to take into account the complex nature of the sample design. Finally, we also not only controlled for individual factors, but considered some community factors as well.

## Conclusions

In summary, this study found a decrease in the HIV prevalence over the studied period and identified two key protective factors: VMMC and education attainment. We found the protective effect of MC both at individual and community levels as the continued increase in the number of males who are circumcised has greatly reduced the number of HIV infections over the past 10 y. With the emergency of pandemics such as COVID-19 and the increase in non-communicable diseases in Eswatini that has prompted donors to shift their focus away from HIV and TB, interventions like VMMC could be a cost-effective strategy. Furthermore, this study revealed that males with a low education level have greater vulnerability to HIV and this vulnerability increased over the studied period. Therefore, future prevention strategies should target these communities, as higher levels of education appear to be protective against HIV seropositivity. This education gradient in the fight against HIV over the years clearly shows that the focus should be more directed on those with lower education as they seem to be still more exposed to the virus than the educated ones.

## Data Availability

None.

## Appendix

**Appendix 1.** OR and 95% CIs of multilevel random intercept logit models predicting positive for HIV among the sexually active adult males, 2006–2007 Swaziland (Eswatini) Demographic and Health Survey (EDHS) and 2016 Eswatini HIV Incidence Measurement Survey (SHIMS 2)

	HIV-positive			
	2006–2007 EDHS		SHIMS 2	
	OR	95% CI	OR	95% CI
**Community-level variables**				
VMMC norm^†^ (reference = median)				
Low: 0%	1.29*	(1.02, 1.66)	1.03	(0.81, 1.30)
High: >10%	1.01	(0.74, 1.36)	0.59	(0.18, 1.98)
**Sexual practices**				
Male circumcision	0.67*	(0.47, 0.94)	0.44**	(0.34, 0.57)
Used a condom at last sex	1.45**	(1.18, 1.80)	2.52**	(2.02, 3.14)
Lifetime number of sexual partners (reference = 1–2)				
3–4	2.57**	(1.83, 3.60)	1.15	(0.82, 1.61)
5–9	2.71**	(1.94, 3.79)	1.48*	(1.06, 2.08)
10 and above	4.92**	(3.46, 6.99)	2.22**	(1.55, 3.19)
Unknown status	2.70**	(1.71, 4.26)	1.62	(1.09, 2.41)
Age at first sex 15 y old or younger	0.70*	(0.52, 0.96)	0.98	(0.69, 1.40)
**Individual/household socioeconomic status (SES)**				
Education attainment (reference = tertiary)				
No education	1.40	(0.89, 2.19)	2.91**	(1.51, 5.59)
Primary education	1.77**	(1.21, 2.59)	4.90**	(3.08, 7.80)
Secondary education	1.70*	(1.16, 2.48)	3.93**	(2.46, 6.28)
High school	1.46	(0.99, 2.14)	3.06**	(1.94, 4.83)
Household poverty	1.17	(0.90, 1.53)	1.51	(0.91, 1.449)
*Background controls*				
Age (y)	1.03**	(1.01, 1.04)	1.12*	(1.10, 1.13)
Marital status (reference = single)				
Married/cohabiting	1.92**	(1.47, 2.51)	1.56**	(1.21, 2.02)
Divorced/separated/widowed	3.64**	(2.44, 5.43)	2.08**	(1.37, 3.17)
Rural residence (reference = urban residence)	0.78	(0.61, 1.01)	0.88	(0.68, 1.15)
Regions (reference = Hhohho)				
Shiselweni	0.86	(0.62, 1.19)	0.93	(0.65, 1.34)
Lubombo	0.80	(0.60, 1.07)	1.12	(0.82, 1.53)
Manzini	0.83	(0.62, 1.11)	0.83	(0.62, 1.12)

^§^ *p*<0.10; **p*<0.05; ***p*<0.01.

^†^The VMMC norm was computed by averaging all surveyed men aged 15–49 y and measuring the proportion of early male circumcision that had occurred at ages 13 y or younger among these men within the communities.

**Appendix 2.** Participant characteristics [percentage or mean (SD)] and year of the survey by sample characteristics among the sexually active adult males who took part in Swaziland (Eswatini) Demographic and Health Survey (EDHS, 2006–2007, N=2470) and Eswatini HIV Incidence Measurement Survey (SHIMS 2, 2016, N=2618)

	EDHS		SHIMS 2, 2016	
	Total sample	2006–2007	2016	*p*-value
Male circumcision (%)	18.61	9.96	26.78	<0.0001
Early circumcision at age 13 y or younger (%)	5.54	6.15	4.97	0.064
**Sexual behavior characteristics**				
Lifetime number of sexual partners				
1–2	41.37	24.13	57.64	
3–4	18.69	24.33	13.37	
5–9	18.51	26.15	11.31	<0.0001
10 and above	13.86	18.87	9.13	
Unknown status	7.57	6.52	8.56 |	
Age at first sex 15 y old or younger (%)	13.25	15.02	11.57	<0.0001
Used a condom at last sex (%)	48.98	41.46	56.07	<0.0001
**Sociodemographic characteristics**				
Age [mean (SD), range: 15–49]	30 (8.94)	29.97 (8.94)	31.09 (8.68)	<0.0001
Education attainment (%)				
No education	6.76	10.28	3.44	
Primary	29.05	30.77	27.43	
Secondary	25.31	25.55	25.10	<0.0001
High school	28.20	23.04	33.08	
Tertiary	10.67	10.36	10.96	
Household poverty (%)	34.43	27.81	40.68	<0.0001
Marital status (%)				
Single	53.33	51.01	55.52	
Married/cohabiting	40.44	42.15	38.83	
Divorced/separated/widowed	6.23	6.84	5.65	<0.001
Urban residence (%)	32.59	38.38	27.12	<0.0001
Regions (%)				
Hhohho	28.60	25.02	31.97	
Lubombo	24.88	28.70	21.28	
Manzini	25.14	18.34	31.55	<0.0001
Shiselweni	21.38	27.94	15.20	
HIV-positive prevalence (%)	26.71	27.80	24.65	0.274
